# Automated H-Scoring in Muscle-Invasive Bladder Cancer IHC: An Internal Validation Study

**DOI:** 10.3390/diagnostics16111673

**Published:** 2026-05-29

**Authors:** Matthew Yap, Ioana-Maria Mihai, Maram Awadh A. Alanazi, Gheorghe-Emilian Olteanu, Alberto Contreras-Sanz, Peter Black, Gang Wang

**Affiliations:** 1 Pathology and Laboratory Medicine, University of British Columbia, Vancouver, BC V6T 1Z7, Canada; 2 Pathology and Laboratory Medicine, BC Cancer, Vancouver, BC V5Z 4E6, Canada; 3 Anatomic Pathology, King Salman Specialist Hospital, Hail 55471, Saudi Arabia; 4 Cellular Pathology Department, Royal Brompton Hospital, London SW3 6NP, UK; 5 Department of Urologic Sciences, University of British Columbia, Vancouver, BC V6H 3Z6, Canada

**Keywords:** digital pathology, H-score, muscle-invasive bladder cancer, biomarker, QuPath, StarDist, automated IHC scoring, cytoplasmic staining, membranous staining

## Abstract

**Background:** Immunohistochemistry (IHC) plays a central role in subtyping of muscle-invasive bladder cancer (MIBC), yet conventional semi-quantitative scoring lacks objectivity and scalability. Automated digital pathology offers potential solutions, but requires robust, marker-specific validation against expert consensus scoring. **Methods:** We developed and internally validated an automated digital pathology pipeline for continuous IHC H-score quantification using QuPath (v0.6.0-arm64). Tissue microarrays (TMAs) were generated from transurethral resection of bladder tumor (TURBT) specimens from a cohort of patients with MIBC treated at Vancouver General Hospital. Cell detection was performed using StarDist (v0.9), followed by automated intensity-based H-score calculation for four epithelial IHC marker stains (CK14, CK20, CK5/6, and Uroplakin II). H-scoring was then restricted to tumor epithelium by object-level classification using a supervised tumor/non-tumor classifier trained on pathologist-reviewed annotations. Automated scores were compared with consensus scores from three blinded pathologists using Pearson correlation, linear regression, intraclass correlation coefficients (ICC), and Bland–Altman analysis. **Results:** Automated H-scores demonstrated strong agreement with pathologist consensus across all four markers. CK14 showed near-perfect agreement (ICC ≈ 0.99) with minimal bias and narrow limits of agreement. CK20 also showed high agreement (ICC ≈ 0.95). CK5/6 and Uroplakin II demonstrated slightly lower agreement (ICC ≈ 0.92 to 0.93) with mild proportional bias. Across markers, the automated pipeline preserved a broad H-score range, with range ratios of 0.96 to 0.99. **Conclusions:** This study establishes a robust, methods-forward pipeline for automated continuous IHC H-scoring in MIBC. The internally validated framework provides a scalable foundation for external cohort testing and future clinical outcome-associated biomarker analyses.

## 1. Introduction

Automated approaches to immunohistochemistry (IHC) quantification in genitourinary (GU) malignancies have been reviewed previously, including in our recent narrative review in *Current Oncology* [1]. Building on this methodological context, the present study implements and internally validates an automated digital pathology pipeline for continuous Histochemical score (H-score) quantification in IHC-stained tissue microarrays derived from a cohort of patients with muscle-invasive bladder cancer (MIBC).

MIBC is a biologically heterogeneous disease in which improved molecular stratification may inform prognosis and therapeutic decision-making, including response to neoadjuvant chemotherapy (NAC) [2,3,4,5,6,7,8]. IHC remains a widely used modality for assessing protein expression patterns reflective of tumor phenotype, including basal and luminal differentiation states [9,10,11,12,13,14]. However, conventional manual H-scoring is inherently subjective, time-intensive, and limited in scalability, especially in TMA-based or retrospective cohort studies [1,15,16,17].

Advances in digital pathology and machine learning have enabled automated quantitative analysis of whole-slide images [18,19,20]. Deep learning approaches for automated H-score estimation have been proposed in selected tumor types, including model-based frameworks such as EndoNet [16,17]. Although feasibility has been demonstrated across multiple platforms, including open-source tools such as QuPath, rigorous validation against expert pathologist scoring remains essential prior to integration into translational research workflows [15,21,22]. In bladder cancer, phenotypic heterogeneity and variability in staining localization across IHC targets highlight the importance of validating automated IHC scoring in a marker-specific manner [3,9,12].

Here, we present the development and internal validation of an automated digital pathology pipeline for continuous IHC H-score quantification in MIBC. Many published studies using QuPath have primarily focused on nuclear biomarker analysis, or binary and ordinal IHC scoring [1]. As such, the novelty of our approach lies in our selection of cytoplasmic- and membranous-staining markers, whose interpretation based on both staining intensity and distribution is more challenging to assess with digital platforms.

Focusing on four epithelial markers relevant to urothelial phenotyping (i.e., two basal markers CK14 and CK5/6, two luminal markers CK20 and Uroplakin II), we evaluate agreement between automated scores and expert pathologist consensus using complementary statistical approaches. This study was designed as a methods-forward pipeline development and internal validation to assess agreement between automated and expert pathologist IHC H-scoring in MIBC. Our study aimed to establish a robust, reproducible, and translationally scalable framework for automated IHC H-score quantification, to support external validation and future outcome-linked biomarker investigations.

## 2. Materials and Methods

### 2.1. Digitized IHC Tissue Microarray Slides and Image Analysis

Tissue microarrays (TMAs) were constructed from transurethral resection of bladder tumor (TURBT) specimens obtained from a retrospective cohort of 42 patients treated with NAC and radical cystectomy at Vancouver General Hospital from 2012 to 2020. The cohort consisted primarily of clinically staged MIBC cases (cT2 to cT4), with one case initially staged as cT1 before surgery but subsequently found at radical cystectomy to have invasive pT3 disease with extension into perivesical soft tissue. The TMAs comprised 84 TURBT tissue-containing cores, with one pair of 1 mm cores sampled per patient. Clinicopathological characteristics of the cohort are summarized in Supplementary Table S1. Each pair of cores was obtained from non-consecutive tumor sections; individual cores were therefore scored independently for downstream analysis.

**Figure 1:**
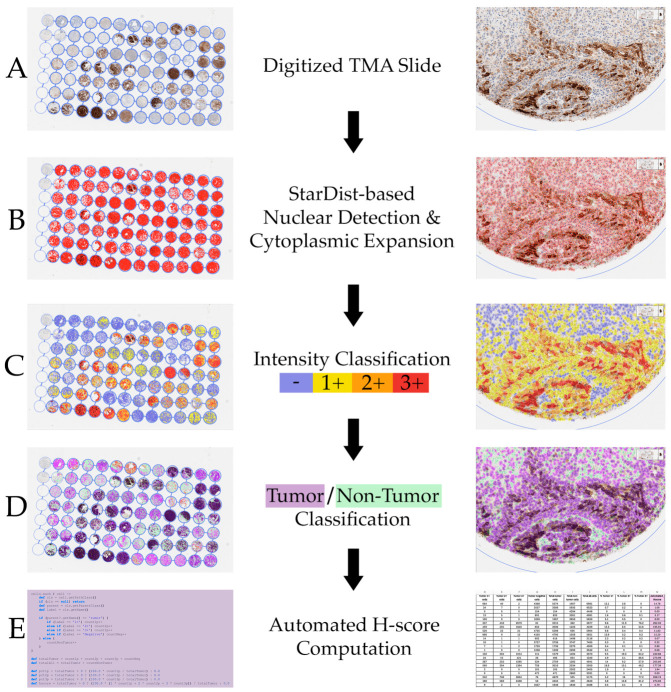
Overview of the pipeline for automated H-scoring of MIBC TURBT TMA cores. (**A**) Digitized TMA slide. (**B**) StarDist-based nuclear detection and cytoplasmic expansion. (**C**) Intensity classification. (**D**) Tumor/Non-Tumor classification. (**E**) Automated H-score computation.

IHC staining for CK5/6, CK14, CK20, and Uroplakin II was performed according to standardized protocols at BC Cancer Pathology. The following monoclonal antibodies were used for immunostaining: CK5/6 (clone D5/16B4, prediluted; Dako, Santa Clara, CA, USA), CK14 (clone LL002, 1:40 dilution; Novocastra, Newcastle upon Tyne, UK), CK20 (clone Ks20.8, prediluted; Dako), and Uroplakin II (clone BC-21, 1:100 dilution; Biocare Medical, Pacheco, CA, USA). Briefly, 4-μm-thick sections were deparaffinized in xylene and hydrated through graded alcohols. Immunostaining was performed using a Dako autostainer (Agilent Technologies, Santa Clara, CA, USA). Slides were incubated with the primary antibody and subsequently with a visualization reagent consisting of secondary goat anti-mouse immunoglobulin and horseradish peroxidase linked to a dextran polymer backbone. The slides were then rinsed with distilled water, incubated with a 3,3-diaminobenzidine (DAB) substrate-chromogen solution, and counterstained with Mayer’s hematoxylin. Stained slides were digitized at 40× magnification using an Aperio AT2 scanner (Leica Biosystems, Vista, CA, USA) and imported into QuPath (v0.6.0-arm64; Queen’s University Belfast, Belfast, Northern Ireland, UK) for image analysis (Figure 1A).

Within QuPath, TMA cores were identified using automated dearraying, followed by visual review and adjustment where cores were not centered. Color deconvolution was performed using estimated stain vectors derived from representative regions on each slide to separate hematoxylin and DAB channels prior to quantitative analysis. No tissue-containing cores were excluded a priori based on poor tissue quality or artifact presence. Instead, non-informative regions, such as cautery artifacts, tissue edges, stromal elements, and areas lacking viable tumor cells, were incorporated into downstream annotations during classifier training to enable automated exclusion during digital analysis.

### 2.2. IHC Marker Selection

Markers were selected to support methodological development and internal validation of an automated pipeline for continuous H-score quantification, focusing on cytoplasmic and membranous-staining markers that are conventionally scored using semi-quantitative, intensity-based schemes in routine practice, to enable direct comparisons between automated and expert H-scores. Bladder cancer markers that primarily exhibit nuclear localization, such as the luminal marker GATA binding protein 3 (GATA3), and/or use alternative scoring schemes were excluded because they were beyond the scope of the automated intensity-weighted cytoplasmic/membranous H-scoring workflow evaluated in this study [13,14,23,24].

Among IHC markers that exhibit predominantly cytoplasmic and membranous staining, we selected two basal-associated markers, CK14 and CK5/6, and two luminal/urothelial-associated markers, CK20 and Uroplakin II. These markers were chosen because they are known to exhibit distinct intensities, dynamic ranges, localization patterns, and degrees of intratumoral heterogeneity [9,10,11,13,14].

### 2.3. Cell Detection

Cell detection was performed in QuPath (v0.6.0-arm64) using StarDist-based nuclear segmentation, a deep-learning approach that represents nuclei as star-convex polygons [22,25]. The detection script was initially developed and tested on heterogeneous single TMA cores to verify parameter behavior and was subsequently generalized to batch-process all cores within each slide. A pretrained StarDist model (v0.9) was applied with percentile normalization (5th to 95th), detection threshold of 0.45, pixel size of 0.5 µm, and cytoplasmic expansion of 4 µm.

Nuclei were automatically filtered by area to exclude debris and merged objects, retaining detections within a range of 5 to 285 µm^2^. Shape and intensity measurements were extracted for each detected cell object. Cell detection was performed across all tissue-containing regions, and was not restricted to tumor areas, to allow downstream cell object classification into either tumor or non-tumor, after intensity classification (Figure 1B).

### 2.4. Intensity Classification and Threshold Calibration

DAB intensity was quantified using the mean cytoplasmic DAB measurement for each detected cell object. Automated intensity classification was performed using fixed DAB intensity thresholds corresponding to “negative”, “1+”, “2+”, and “3+” classes, resulting in intensity labels assigned to all cell detections prior to tumor/non-tumor classification. Thresholds were calibrated with a single pathologist who was not involved in subsequent blinded consensus scoring by three other pathologists. Threshold classes were assigned to 40 randomly selected regions of variable DAB intensity across the four TMA slides, and average DAB stain measurements per intensity class were incorporated into the Groovy script for automated intensity thresholding after cell detection (Figure 1C).

### 2.5. Tumor/Non-Tumor Annotation and Classification

Supervised tumor and non-tumor classification was performed using an object classifier trained with manually annotated regions under the supervision of one pathologist. Annotation classes included tumor epithelium (*n* = 676), non-tumor tissue (including stroma and immune cells; *n* = 322), background or staining artifact (*n* = 15), and “ignore” regions capturing non-informative areas, tissue edges, and cautery artifacts (*n* = 103).

Classifier training was performed using annotations generated on a subset of 15 cores from the Uroplakin II TMA slide, which facilitated precise annotation of tumor boundaries, due to enhanced visual delineation of cell membranes and urothelial morphology. The resulting object classifier was generalized and applied to all 84 tissue-containing cores across CK14, CK5/6, CK20, and Uroplakin II slides (Figure 1D).

### 2.6. Automated H-Score Calculation

Intensity classification assigned each detected cell object to one of four categories (negative, 1+, 2+, or 3+) based on calibrated cytoplasmic DAB measurements, while tumor/non-tumor classification identified tumor cells for analysis. Automated H-score computation was performed after dual classification, using cell objects that retained both tumor class and intensity class (i.e., tumor: negative, tumor: 1+, tumor: 2+, tumor: 3+). All detection objects that inherited “non-tumor”, “background”, or “ignore” classes were excluded from H-score computation. For each TMA core, tumor-classified cell objects were aggregated by intensity category. Automated H-score computation yielding a continuous range from 0 to 300 was implemented by a Groovy script in QuPath according to the following cell count-based formula:

(1) $$H‐\mathrm{score}\;=\;\frac{\left(1\;\times \;N_{tumor:\;1+}\right)+\left(2\;\times \;N_{tumor:\;2+}\right)+\left(3\;\times \;N_{tumor:\;3+}\right)}{N_{total\;tumor\;cell\;objects}}\times 100,$$

which is mathematically equivalent to the intensity-weighted formula:

(2) $$H‐\mathrm{score}\;=\;(1\times \%\;tumor\;1^{+})+(2\times \%\;tumor\;2^{+})+(3\times \%\;tumor\;3^{+}),$$

in which percentages represent the proportion of tumor cell objects within each intensity class, relative to the total number of tumor-classified cell objects. Per-core H-scores per TMA slide were batch-exported to Microsoft^®^ Excel for downstream analysis (Figure 1E).

### 2.7. Consensus Scoring and Statistical Analyses

Conventional visual H-scoring (from a scale of 0 to 300) on 84 tissue-containing cores per IHC TMA slide was independently performed by three pathologists who were blinded to the automated scoring results, and uninvolved in automated intensity threshold calibration. A consensus H-score per core was calculated as the mean of the three expert-assessed H-scores. Automated H-scores were statistically compared against the consensus scores.

Agreement between automated and consensus H-scores was evaluated using Pearson correlation, linear regression, intraclass correlation coefficient (ICC), and Bland–Altman analysis. Pearson correlation and linear regression were used to quantify the linear association between scoring methods. ICC was used to evaluate absolute agreement under a two-way mixed-effects model [26,27]. Bland–Altman analysis characterized bias and dispersion across the continuous range from 0 to 300 [28].

All statistical analyses were performed in R (v4.5.0; R Foundation for Statistical Computing, Vienna, Austria) using *ggplot2* and *psych* packages. ICC(3,1) values were calculated using the *psych* package (two-way mixed-effects model, absolute agreement, single measurement). Bland–Altman bias and limits of agreement were computed using base R functions and visualized using *ggplot2* to assess systematic bias across the continuous H-score range. Statistical significance was defined as *p* < 0.05.

## 3. Results

### 3.1. Cell Segmentation Performance

**Figure 2:**
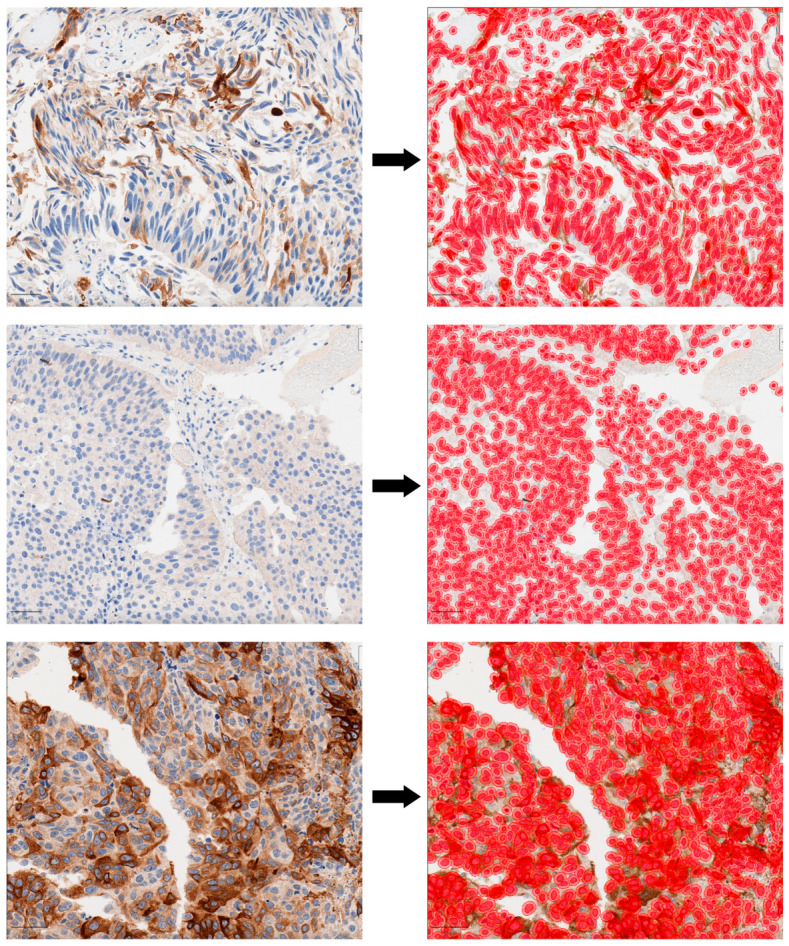
StarDist-based segmentation across morphologically heterogeneous MIBC tissue. **Left**: examples of hematoxylin- and DAB-stained tumor regions. **Right**: corresponding StarDist-based segmentation outputs.

StarDist-based cell segmentation enabled delineation of individual cell boundaries around hematoxylin-stained nuclei across heterogeneous MIBC tumor tissue. Segmentation boundaries corresponded closely to nuclear contours across the diverse tumor architectures present in the TURBT specimens. Global segmentation produced discrete cell objects in both tumor nests and stromal regions, enabling subsequent object-level classification and downstream analysis (Figure 2).

### 3.2. Automated Intensity-Thresholding and Classification of Tumor Cell Objects

**Figure 3:**
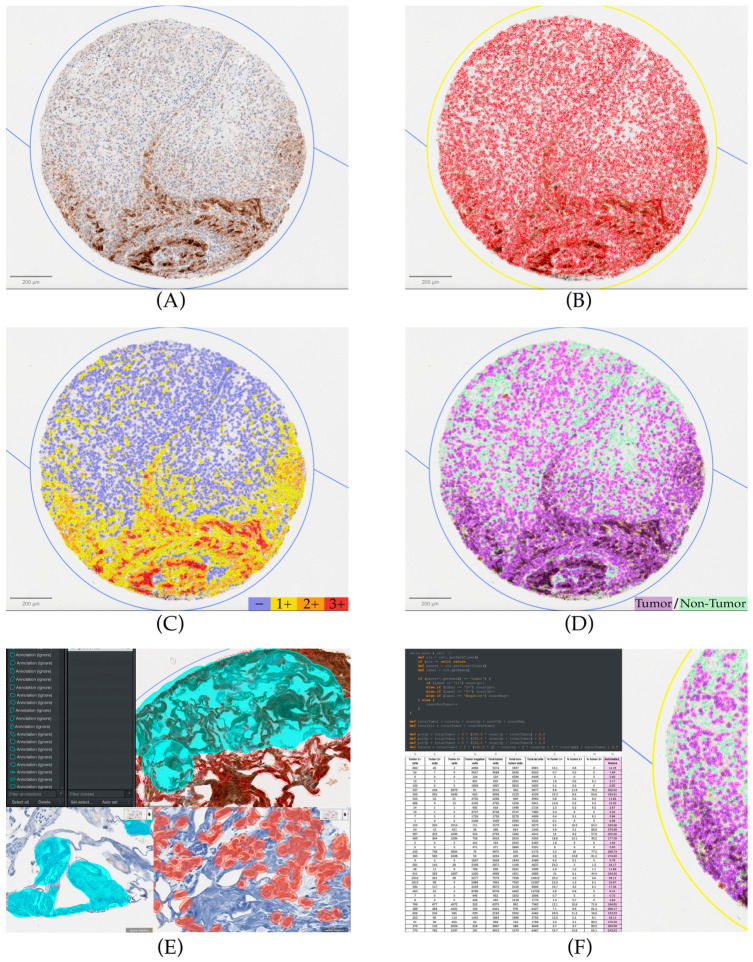
Automated Object-level Classification and H-score computation in QuPath. (**A**) Example of a Uroplakin II IHC TMA core. (**B**) Cell objects from StarDist-based cell segmentation. (**C**) Cytoplasmic DAB mean intensity classification of detected cell objects into “negative”, “1+”, “2+”, and “3+”. (**D**) Tumor/Non-Tumor object classification used to identify tumor areas for inclusion in computation. (**E**) Automated exclusion of artefactual or non-informative regions due to prior classifier training. (**F**) Cell object-count-based scoring batch-exported from QuPath.

The automated pipeline successfully performed dual classification of cell objects across all 84 cores for each marker. Intensity-thresholding of cell detections based on calibrated cytoplasmic DAB measurements resulted in visual stratification of cell objects into intensity classes (Figure 3C). Tumor/non-tumor classification at the cell-object level showed delineation of tumor epithelium from stromal, inflammatory, and artifactual regions (Figure 3D). Non-informative regions (e.g., cautery artifact, tissue edges, and areas of tissue loss) were successfully excluded through background and ignore classes incorporated during classifier training (Figure 3E). These steps produced tumor-classified, intensity-labeled cell objects for downstream H-score computation (Figure 3F).

### 3.3. Agreement Between Automated and Pathologist H-Scores

#### 3.3.1. Automated H-Scoring Performance: CK14

**Figure 4:**
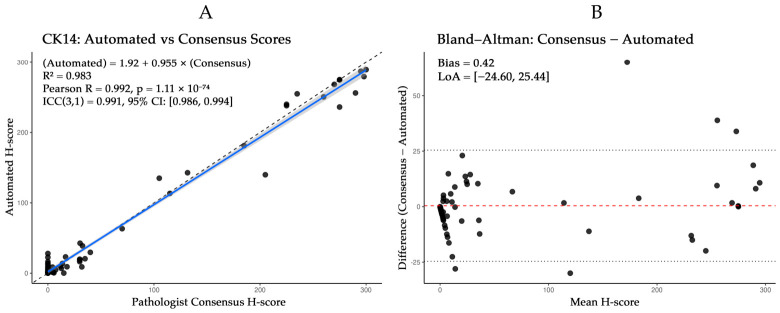
Agreement between automated and pathologist consensus H-scores for CK14. (**A**) Scatter plot of automated vs. consensus H-scores with fitted linear regression and identity line (y = x). (**B**) Bland–Altman plot demonstrating mean difference (bias) and 95% limits of agreement.

Automated CK14 H-scores demonstrated near-perfect agreement with pathologist consensus H-scores (Figure 4). Pearson correlation was high (R = 0.992; R^2^ = 0.983), indicating a very strong linear association between automated and consensus scores. The regression slope was 0.956, suggesting minimal proportional bias relative to the identity line (y = x). The ICC(3,1) was 0.991 (95% CI 0.986 to 0.994), reflecting excellent absolute agreement between automated and consensus H-scores. Bland–Altman analysis demonstrated minimal mean bias (0.42) with narrow limits of agreement.

#### 3.3.2. Automated H-Scoring Performance: CK20

**Figure 5:**
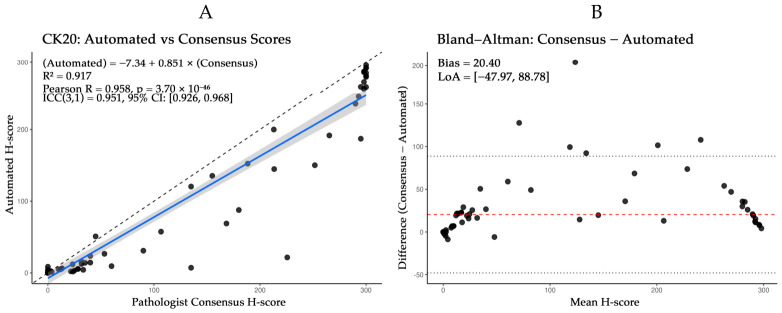
Agreement between automated and pathologist consensus H-scores for CK20. (**A**) Scatter plot of automated vs. consensus H-scores with fitted linear regression and identity line (y = x). (**B**) Bland–Altman plot demonstrating mean difference (bias) and 95% limits of agreement.

Automated CK20 H-scores demonstrated strong agreement with expert consensus H-scores (Figure 5). Pearson correlation was high (R = 0.958; R^2^ = 0.917), indicating a strong linear association between automated and consensus scores. The regression slope was 0.851 with a negative intercept (−7.34), reflecting constant, proportional downward deviation from the identity line, consistent with modest systematic underestimation by the automated method. The ICC(3,1) was 0.951 (95% CI 0.926 to 0.968), indicating excellent absolute agreement overall. Bland–Altman analysis demonstrated a positive mean bias of 20.40 with wider limits of agreement (−47.97 to 88.77).

#### 3.3.3. Automated H-Scoring Performance: CK5/6

**Figure 6:**
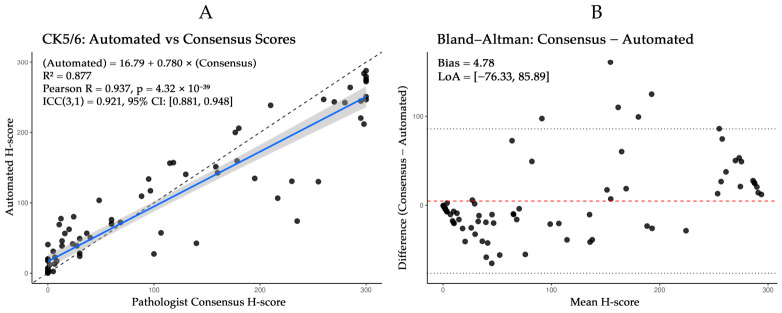
Agreement between automated and pathologist consensus H-scores for CK5/6. (**A**) Scatter plot of automated vs consensus H-scores with fitted linear regression and identity line (y = x). (**B**) Bland–Altman plot demonstrating mean difference (bias) and 95% limits of agreement.

Automated CK5/6 H-scores demonstrated strong overall agreement with expert consensus scores (Figure 6). Pearson correlation was high (R = 0.937; R^2^ = 0.877), indicating a strong linear association between automated and consensus scores. The regression slope was 0.780 with an intercept of +16.80, reflecting proportional deviation from the identity line (y = x), with relative overestimation at lower H-scores and underestimation at higher H-scores. The ICC(3,1) was 0.921 (95% CI 0.881 to 0.948), indicating good absolute agreement. Bland–Altman analysis demonstrated a mean bias of 4.78 with wider limits of agreement (−76.32 to 85.89).

#### 3.3.4. Automated H-Scoring Performance: Uroplakin II

**Figure 7:**
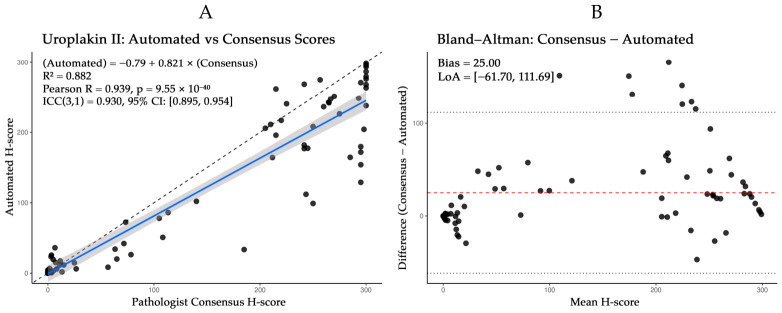
Agreement between automated and pathologist consensus H-scores for Uroplakin II. (**A**) Scatter plot of automated vs. consensus H-scores with fitted linear regression and identity line (y = x). (**B**) Bland—Altman plot demonstrating mean difference (bias) and 95% limits of agreement.

Automated Uroplakin II H-scores showed strong agreement with expert consensus H-scores (Figure 7). Pearson correlation was high (R = 0.939; R^2^ = 0.882), indicating a strong linear association between automated and consensus scores. The regression slope was 0.821 with a near-zero intercept, reflecting proportional deviation from the identity line (y = x) with increasing underestimation at higher H-scores. The ICC(3,1) was 0.931 (95% CI 0.895–0.954), indicating excellent absolute agreement. Bland–Altman analysis demonstrated a positive mean bias of 24.99 with wider limits of agreement (−61.70 to 111.69).

### 3.4. Preservation of H-Score Dynamic Range

Automated H-scores preserved a broad dynamic range across markers. Minimum and maximum values closely approximated those observed in pathologist consensus scores, with automated-to-consensus range ratios between 0.96 and 0.99 (Table 1). Supplementary Figure S1 illustrates the distribution of H-score values across TMA cores.

## 4. Discussion

In this study, we developed and internally validated an automated digital pathology pipeline for continuous IHC H-score quantification in MIBC. Our pipeline showed that automated scoring can closely reproduce expert consensus across multiple epithelial differentiation markers. The analytical framework we developed therefore supports a generalizable approach for automated H-scoring of cytoplasmic epithelial markers, an area that remains relatively underexplored in automated IHC quantification.

Most prior work in automated IHC analysis has focused on markers such as Ki-67 and HER2, whose binary and ordinal scoring demonstrate greater compatibility with region- or boundary-based algorithms [1]. In contrast, fewer studies have focused on intensity-weighted scoring for markers that exhibit cytoplasmic localization [1]. Cytoplasmic staining poses distinct challenges for automation, including diffuse spatial distribution, variable intracellular localization, and dependence on accurate cell segmentation. Signal quantification may also be affected by cell size heterogeneity, imperfect boundary delineation, and non-uniform intracellular staining distribution.

In our pipeline, intensity thresholding was based on mean cytoplasmic DAB measurements of cell objects. This strategy was selected to enable a consistent measurement approach across markers but may dilute and underestimate DAB intensity for markers with stronger membranous localization when averaged across the expanded cytoplasmic measurement regions. These challenges likely contributed to the variability in agreement observed across markers in our results. Future optimization of the pipeline may incorporate membrane-aware or compartment-specific measurement regions to improve quantification of markers with intense membranous staining.

Agreement between automated and pathologist scoring was assessed using continuous measurement metrics rather than threshold-based classification. Within this analysis, CK14 and CK20, which showed predominantly cytoplasmic staining, demonstrated near-perfect agreement with pathologist consensus, whereas CK5/6 and Uroplakin II, which showed both cytoplasmic and membranous staining, exhibited slightly greater variability. Such variability reflects known differences in staining localization and expression patterns reported for these markers in urothelial carcinoma [9,12]. These findings highlight the importance of marker-specific evaluation when implementing automated digital pathology workflows.

Moreover, we found that automated H-scores notably preserved a broad measurement range across markers, closely approximating the dynamic range observed in pathologist consensus scoring (Table 1; Figure S1). These findings demonstrate a key advantage of automated IHC quantification over conventional manual scoring, which frequently relies on categorical or semi-quantitative scales that compress biologically meaningful variation into a limited number of ordinal classes [19], potentially limiting sensitivity in detecting biologically relevant differences between tumors [19,20].

A limitation of this study is the use of a single-institution retrospective TMA cohort of 42 patients and a focused four-marker panel, which was selected for methodological development and internal validation rather than comprehensive molecular subtyping. Evaluation in additional TMA cohorts will be required to assess external validity across independent staining batches, tissue cohorts, and clinical contexts, and to further define robustness across laboratories and pre-analytical conditions. Such studies represent an important next step toward broader adoption of automated digital pathology-based quantification methods in biomarker-driven precision oncology [19,20].

Future studies may also extend this framework to additional marker classes, including mesenchymal and stromal markers such as Vimentin, α-smooth muscle actin (α-SMA), and Desmin, given the recognized stroma-rich/mesenchymal phenotype in a subset of MIBC [3,29,30]. Such markers would require compartment-aware or region-based adaptations of the current pipeline, particularly where staining signals localize to stromal rather than epithelial tumor compartments.

Beyond technical external validation, application of this pipeline to larger cohorts will enable investigation of associations between automated IHC features and clinically relevant outcomes, including molecular subtype classification and response to therapy. Continuous digital H-score measurements may also support the development of clinically actionable classification thresholds once sufficiently large datasets and outcome-associated cohorts become available. Integration of digital pathology measurements with genomic and clinical data may further enable biomarker-driven analytical frameworks and machine learning-based predictive modeling in bladder cancer research.

## 5. Conclusions

This study demonstrates that automated digital pathology analysis can reproduce expert pathologist H-score assessments with high agreement across multiple epithelial differentiation markers in MIBC. The internally validated pipeline provides a reproducible framework for continuous IHC quantification that preserves the dynamic range of staining intensity while enabling scalable analysis of TMA datasets. With external validation in independent cohorts, automated H-scoring may support future biomarker discovery and outcome-linked analyses in bladder cancer and other GU tumors.

## Figures and Tables

**Table 1:** Dynamic Range and Distribution of Automated and Consensus H-Scores (*n* = 84 cores per marker).

H-Score Metric	CK14	CK20	CK5/6	Uroplakin II
Minimum (Automated)	0	0	0	0
Maximum (Automated)	289	296	288	298
* Range Ratio	0.96	0.99	0.96	0.99
Median (Consensus, IQR)	0.0 [0.0–32.2]	22.5 [0.0–182.1]	33.3 [0.0–183.8]	106.7 [3.3–264.8]
Median (Automated, IQR)	4.8 [0.9–28.4]	5.3 [0.3–124.2]	57.0 [10.7–152.5]	61.6 [3.4–219.3]

* Range ratio = (Automated max−Automated min) ÷ (Consensus max−Consensus min). Consensus H-scores range from 0 to 300 by definition.

## Data Availability

The data presented in this study are available on request from the corresponding author. Restrictions apply due to ethical requirements regarding patient data sharing.

## Supplementary Materials

The following supporting information can be downloaded at: https://www.mdpi.com/article/10.3390/diagnostics16111673/s1. Figure S1. Heatmap of Automated and Expert Consensus H-score values across individual TMA cores per IHC marker slide; Cell Object Counts and Raw H-Score Data; Table S1. Clinicopathological characteristics of the 42-patient MIBC TURBT TMA cohort.

## Abbreviations

The following abbreviations are used in this manuscript:

α-SMA	Alpha Smooth Muscle Actin
CI	Confidence Interval
CK	Cytokeratin
DAB	3,3′-Diaminobenzidine
GATA3	GATA binding protein 3
GU	Genitourinary
H-score	Histochemical Score
ICC	Intraclass Correlation Coefficient
IHC	Immunohistochemistry
MIBC	Muscle-Invasive Bladder Cancer
NAC	Neoadjuvant Chemotherapy
TMA	Tissue Microarray
TURBT	Transurethral Resection of Bladder Tumor
