# Supplementary material for: Automated H-Scoring in Muscle-Invasive Bladder Cancer IHC: An Internal Validation Study
Source: Diagnostics (Basel). 2026 May 29;16(11):1673. doi: 10.3390/diagnostics16111673 (PMC13256710; doi:10.3390/diagnostics16111673)
Supplement: Supplementary file 1 [file diagnostics-16-01673-s001.zip › Supplementary Figure 1.pdf]

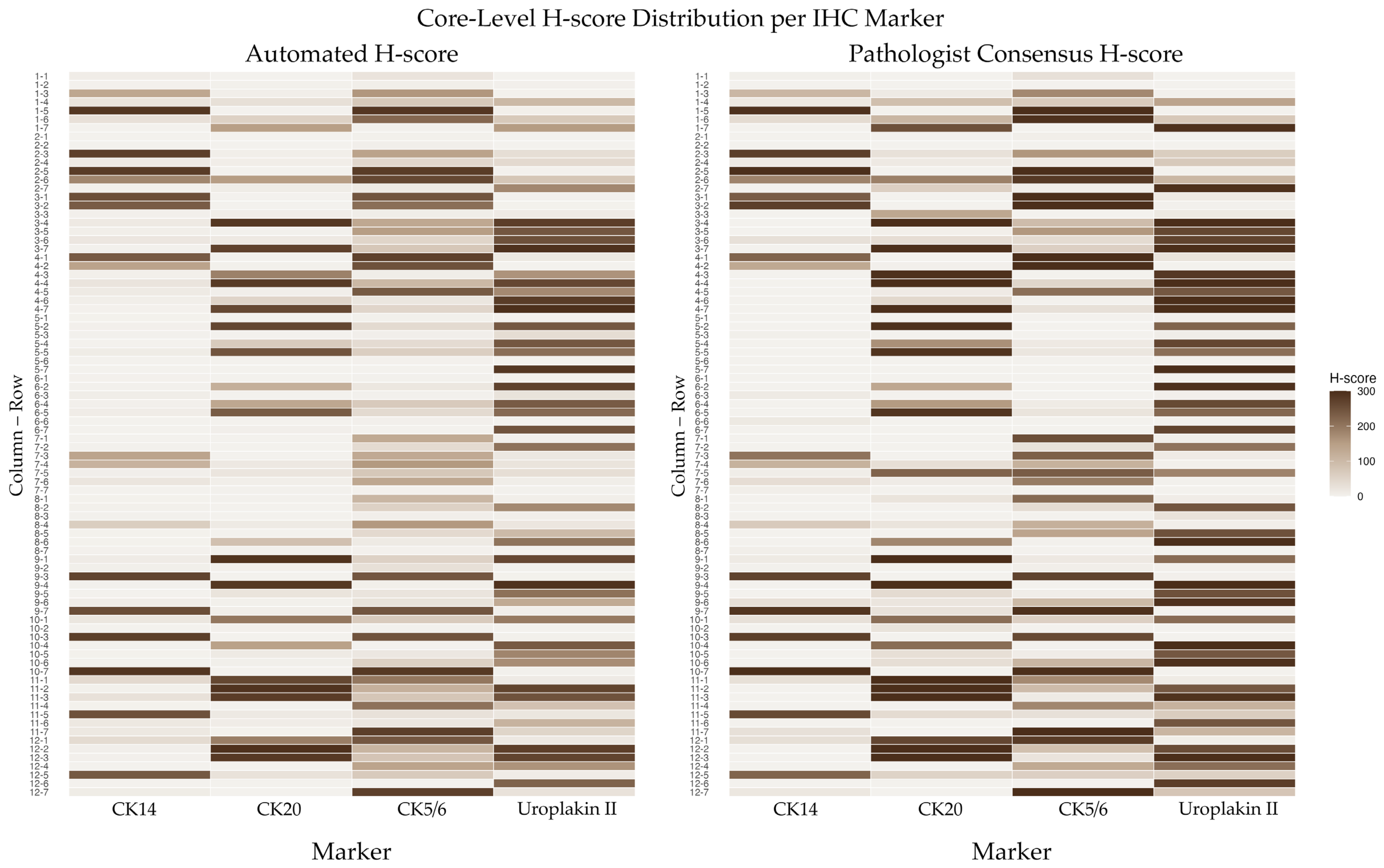

Figure S1. Heatmap of Automated and Expert Consensus H-score values across individual TMA cores per IHC marker slide. Rows correspond to individual cores labeled by TMA column and row number, and columns correspond to the four evaluated markers (CK20, CK14, CK5/6 and Uroplakin II). Colour intensity represents H-score value on a scale from 0 to 300.
(A) Automated H-Scores; (B) Pathologist Consensus H-Scores.
