# Supplementary material for: Automated H-Scoring in Muscle-Invasive Bladder Cancer IHC: An Internal Validation Study
Source: Diagnostics (Basel). 2026 May 29;16(11):1673. doi: 10.3390/diagnostics16111673 (PMC13256710; doi:10.3390/diagnostics16111673)
Supplement: Supplementary file 1 [file diagnostics-16-01673-s001.zip › Supplementary Table S1.pdf]

Supplementary Table S1. Clinicopathological characteristics of the 42-patient MIBC TURBT TMA cohort.

Values are shown as n (%) unless otherwise indicated. Percentages were calculated using n = 42, except where information was missing. Pathologic T and N stages refer to radical cystectomy specimens after neoadjuvant chemotherapy. One case was clinically staged as cT1 before surgery but was found at radical cystectomy to have invasive pT3 disease with extension into perivesical soft tissue.

**Cohort size**	
Patients	42
TURBT TMA cores	84
Age at radical cystectomy, median (range), years	62.5 (36–77)
**Sex**	
Male	32 (76.2%)
Female	10 (23.8%)
**Clinical T stage**	
cT1	1 (2.4%)
cT2	28 (66.7%)
cT3	10 (23.8%)
cT4	3 (7.1%)
**Pathologic T stage at radical cystectomy**	
pT0	14 (33.3%)
pTa	2 (4.8%)
pTis	2 (4.8%)
pT1	1 (2.4%)
pT2	9 (21.4%)
pT3	10 (23.8%)
pT4	4 (9.5%)

**Neoadjuvant chemotherapy regimen**	
Gemcitabine/cisplatin	40 (95.2%)
Gemcitabine/carboplatin	2 (4.8%)
NAC cycles, median (range), n = 41	4 (2–8)
**Primary TURBT histology**	
Urothelial carcinoma	41 (97.6%)
Squamous cell carcinoma	1 (2.4%)
**Clinical N stage**	
cN0	35 (83.3%)
cN1	4 (9.5%)
cN2	1 (2.4%)
cN3	1 (2.4%)
Missing/NA	1 (2.4%)
**Pathologic N stage at radical cystectomy**	
pN0	31 (73.8%)
pN1	5 (11.9%)
pN2	2 (4.8%)
pN3	4 (9.5%)
**Response to neoadjuvant chemotherapy**	
Complete responder	13 (31.0%)
Partial responder	5 (11.9%)
Non-responder	24 (57.1%)
